# Association of lactate to albumin ratio with short-term and long-term mortality in critically ill patients with heart failure complicated by sepsis: a retrospective study using the MIMIC-IV database

**DOI:** 10.3389/fcvm.2025.1636375

**Published:** 2025-09-23

**Authors:** Yuming Wu, Ling Wang, Qiuyan Wen

**Affiliations:** ^1^ Department of Cardiology, Quanzhou First Hospital Affiliated to Fujian Medical University, Quanzhou, China; ^2^ Department of Anesthesiology, The Second Affiliated Hospital of Fujian Medical University, Quanzhou, China

**Keywords:** heart failure, sepsis, MIMIC-IV, lactate to albumin ratio, mortality

## Abstract

**Background:**

Elevated lactate to albumin ratio (LAR) has been associated with poor prognosis in critical illnesses. However, evidence regarding LAR in patients with heart failure (HF) complicated by sepsis remains limited. This study aimed to explore the relationship between LAR and both short-term and long-term mortality in this population.

**Method:**

Patient data were extracted from the Medical Information Mart for Intensive Care (MIMIC-IV) database and stratified into quartiles based on LAR. The primary endpoints were 28-day and 365-day all-cause mortality. Kaplan–Meier survival analysis was performed to compare outcomes across the four groups. Association between LAR and mortality was assessed using restricted cubic splines (RCS) and Cox regression analysis. Additionally, subgroup and sensitivity analyses were conducted.

**Result:**

Among 4,242 participants (mean age 72.04 ± 13.50 years; 57.33% male), Kaplan–Meier analysis showed that higher LAR levels were associated with increased 28-day and 365-day all-cause mortality (log-rank *P* < 0.001). Cox regression analysis confirmed that elevated LAR was independently associated with higher 28-day and 365-day all-cause mortality (HR: 1.101, 95% CI 1.005–1.205; HR: 1.125, 95% CI 1.039–1.218). The highest LAR quartile (>0.97) remained significantly associated with both 28-day and 365-day mortality (Q4 vs. Q1: HR: 1.313, 95% CI 1.063–1.622; HR: 1.310, 95% CI 1.092–1.571). RCS analysis indicated a linear positive correlation between LAR and mortality (*P* for nonlinear > 0.05). Subgroup analysis revealed a significant interaction with hypertension (*P* for interaction = 0.033 for 28-day; *P* for interaction = 0.015 for 365-day). Sensitivity analyses confirmed the robustness of these findings.

**Conclusion:**

In critically ill patients with HF complicated by sepsis, LAR is a reliable and independent predictor of mortality. Patients in the highest LAR quartile (>0.97) have a significantly increased risk of death, providing a clinically useful reference for rapid identification of high-risk individuals. The significant interaction observed in hypertensive subgroups highlights the need for heightened clinical attention. Overall, LAR may serve as a practical biomarker for risk stratification and prognostic evaluation in this vulnerable population.

## 1 Introduction

The growing global burden of cardiovascular disease has become a major challenge to human health. HF is a rapidly growing public health issue with an estimated prevalence of 40 million individuals globally (1, 2). Moreover, sepsis accounts for almost one-quarter of deaths in people with HF (3). Sepsis has been identified as a major cause of non-cardiovascular death and hospitalization in people with HF, including those with preserved and reduced left ventricular ejection fraction (LVEF) (4–6). Therefore, close monitoring and comprehensive treatment of HF patients with severe infections in the intensive care unit (ICU) are critically important.

Patients with HF complicated by sepsis frequently develop acid-base disorders and tissue hypoxia. Serum lactate levels, which increase due to anaerobic metabolism under hypoxic conditions, are an important biomarker for evaluating disease severity (7). Specifically in sepsis, lactate level is a reliable parameter for guiding diagnosis, treatment decisions, and prognosis prediction (8). Additionally, albumin is one of the acute-phase proteins that reflect inflammation severity, nutritional status, and chronic disease status (9). Combining these two parameters has produced a biomarker, the lactate to albumin ratio (LAR), for prognostic assessment. Recent research has proposed LAR as an important prognostic factor for sepsis patients admitted to ICU (10, 11). Furthermore, LAR can predict prognosis in critically ill patients with HF, atrial fibrillation (AF), acute myocardial infarction (AMI) and other cardiovascular diseases (12–15). However, the predictive effect of LAR on mortality in ICU-admitted patients with both HF and sepsis remains largely unknown.

This study aimed to evaluate the prognostic value of LAR for mortality risk in ICU-admitted patients with HF complicated by sepsis. While prior evidence supports the prognostic role of LAR in various critical conditions, its applicability to this high-risk population requires further investigation. Based on this research gap, we hypothesized that LAR would serve as a reliable predictor of both short-term and long-term clinical outcomes in this patient population.

## 2 Method

### 2.1 Source of data

This retrospective study utilized data from the publicly available Medical Information Mart for Intensive Care IV (MIMIC-IV, version 3.1) database. Developed by the Laboratory for Computational Physiology at the Massachusetts Institute of Technology (MIT), received Institutional Review Board (IRB) approvals from both MIT and Beth Israel Deaconess Medical Center (BIDMC). The database contains clinical records spanning 2008 to 2022, encompassing over 70,000 intensive care unit admissions from BIDMC (16). It provides comprehensive clinical data including demographics, laboratory tests, vital signs, surgical procedures, disease diagnoses, pharmaceutical information, and follow-up survival status. As this study used deidentified public data, it was exempt from requiring patient informed consent and additional ethics committee approval. One author (Yuming Wu) completed the required National Institutes of Health (NIH) training on human research participant protection and passed the Collaborative Institutional Training Initiative (CITI) examination, obtaining permission to extract data from MIMIC-IV (Record ID: 14251740).

### 2.2 Cohort selection

In this study, HF was defined according to the Universal Definition and Classification of Heart Failure (17), characterized by typical clinical symptoms and/or signs of HF in combination with objective evidence of structural or functional cardiac abnormalities. In the MIMIC-IV database, patients were identified using validated ICD-9/ICD-10 codes for HF, which have been widely applied in prior large-scale epidemiological studies. Although key indicators such as BNP, NT-proBNP, and LVEF are standard parameters for confirming HF phenotype and classification, these variables had high missing rates (38.5% for LVEF and 76.9% for NT-proBNP). Consequently, HF patients could not be reliably subtyped by ejection fraction. And sepsis was defined according to the Third International Consensus Definitions for Sepsis (Sepsis-3) as those with suspected infection and an acute change of ≥2 points in the total Sequential Organ Failure Assessment (SOFA) score (18).

**Figure 1:**
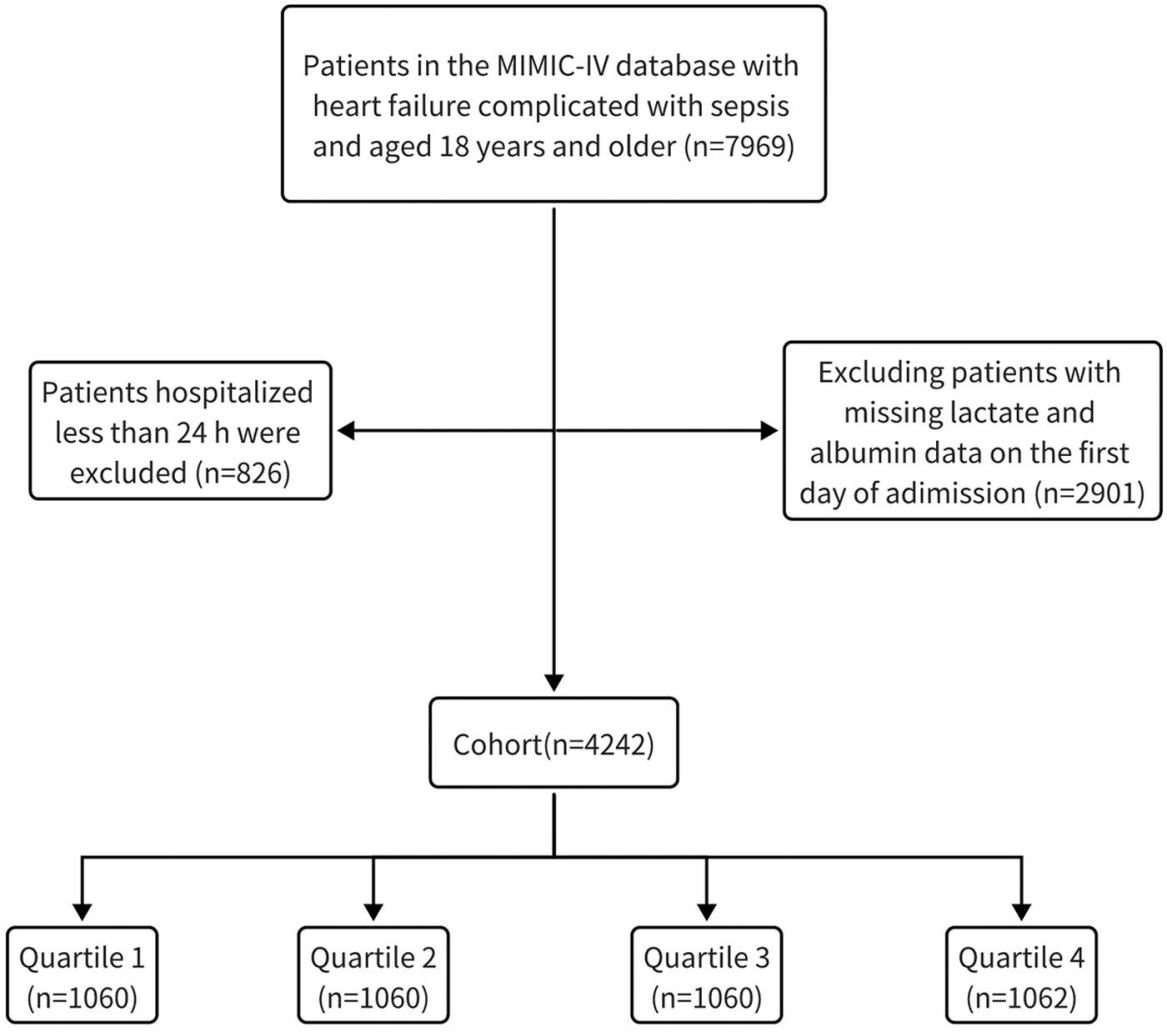
The flow chart of patient selection.

This study focused on the overall HF population as identified by ICD codes, and included patients with HF complicated by sepsis who were admitted to the ICU for the first time and were eligible adult patients over 18 years old. We excluded patients based on the following criteria: (a) those who had recurrent hospitalizations and were not admitted to the ICU for the first time; (b) those with an ICU length of stay less than 24 hours; (c) those missing lactate or albumin data. Ultimately, 4,242 eligible patients were stratified into four groups according to quartiles of the LAR (Figure 1).

### 2.3 Data extraction

All variables were collected within 24 hours of admission. The following data were extracted from the MIMIC-IV database (v3.1). (1) Demographics: Age, sex. (2) Vital signs: heart rate (HR), systolic blood pressure (SBP), diastolic blood pressure (DBP), mean arterial pressure (MAP), respiratory rate (RR), and pulse blood oxygen saturation (SpO_2_). (3) Complications: AF, hypertension, liver disease (LD), chronic kidney disease (CKD), malignant tumor (MT), diabetes, AMI, chronic obstructive pulmonary disease (COPD), and ventricular arrhythmia (VA). (4) Test Indicators: hemoglobin, platelets, red blood cells (RBC), white blood cells (WBC), red cell distribution width (RDW), albumin, lactate, anion gap, calcium, chloride, glucose, potassium, sodium, phosphate, bicarbonate, international normalized ratio (INR), prothrombin time (PT), partial thromboplastin time (PTT), alanine aminotransferase (ALT), aspartate aminotransferase (AST), total bilirubin (TBIL), creatinine (Cr), blood urea nitrogen (BUN), N-terminal pro-B-type natriuretic peptide (NT-proBNP), LVEF, PCO_2_, PO_2_ and pH. (5) Treatments: angiotensin-converting enzyme inhibitors/angiotensin receptor blockers (ACEIs/ARBs), calcium channel blocker (CCB), beta-blockers, diuretics, glucocorticoid (GC) and renal replacement therapy (CRRT). (6) Disease Severity Scores: SOFA, Charlson Comorbidity Index (CCI), Acute Physiology Score III (APSIII), Simplified Acute Physiology Score II (SAPSII), and Oxford Acute Severity of Illness Score (OASIS). (7) Outcomes: 28-day mortality, and 365-day mortality. All test markers were measured at initial admission to the ICU and prior to any treatment. To avoid the inefficiency and potential bias of direct exclusion of cases with missing data, multiple imputation was used to estimate missing variables (19). Variables with missing rates exceeding 20%, specifically NT-proBNP (76.9%) and LVEF (38.5%), were excluded (Supplementary Table S1). Missing values were imputed by using multiple imputation.

### 2.4 Calculation of LAR and outcome

LAR was defined as follows: LAR = lactate (mmol/L)/albumin (g/dl) (20). To assess the short-term and long-term risk of death in patients with HF complicated by sepsis, the primary endpoints of this study were 28-day and 365-day all-cause mortality.

### 2.5 Statistical analysis

For continuous variables with a normal distribution, the baseline data are presented as mean ± SD. For continuous variables with a non-normal distribution, data are presented as median ± interquartile range (IQR). Categorical variables are summarized as frequencies and percentages (%). Comparison of continuous variables were conducted using one-way analysis of variance (ANOVA) or the Kruskal–Wallis test. Categorical variables were compared using the chi-square or Fisher's exact tests and are presented as absolute numbers with percentages.

Firstly, Kaplan–Meier survival analysis was used to estimate the incidence of outcomes, and differences between the groups were assessed by using the log-rank test. Univariable Cox regression analysis was used to assess the association between LAR and 28-day and 365-day mortality. Multivariable Cox models were constructed by including clinically relevant variables or those showing univariate relationships with the outcomes. Baseline and clinically relevant variables with a significance level *P* < 0.05 in the univariable Cox regression analysis were included in the multivariate models for adjustment (Supplementary Table S2). The final model variables were carefully selected based on the number of available events. Multicollinearity was examined using the variance inflation factor (VIF), and the maximum VIF in this study was 5 (Supplementary Table S3). Subsequently, hierarchical Cox regression models were developed with sequential covariate adjustments to examine the independent association between LAR and the outcomes. Model 1 included only LAR. Model 2 was adjusted for age, SBP, RR and SpO_2_. Model 3 was further adjusted for laboratory tests, including WBC, RDW, anion gap, chloride, potassium, phosphate, bicarbonate, PTT, AST, TBIL, Cr, BUN, PCO_2_, PO_2_ and pH. Model 4 additionally incorporated AF, hypertension, LD, CKD, MT, diabetes, AMI, COPD, VA, ACEIs/ARBs, CCB, beta-blockers, diuretics, vasopressor, GC, CRRT, SOFA, CCI, APSIII, SAPSII, OASIS. Subgroup analysis was performed to evaluate whether the effect of LAR on survival time differed across subgroups. Finally, LAR was also analyzed as a continuous variable using RCS to clarify dose-response associations with outcome risk. Sensitivity analysis was performed to determine the robustness of the findings.

Data processing and analysis were performed using DecisionLinnc v1.0.9. DecisionLinnc is a platform that integrates multiple programming language environments and enables data processing, data analysis, and machine learning through a visual interface (21). Statistical significance was set at *P* < 0.05.

## 3 Results

### 3.1 Baseline characteristics of study individuals

A total of 4,242 patients met the inclusion criteria and were included in the analysis. The mean age of the enrolled patients was 72.04 ± 13.50 years old, and 57.33% were male. The 28-day and 365-day mortality rates were 22.91% and 30.13%, respectively. Based on the quartiles of LAR (Q1: <0.41, Q2: 0.41–0.61, Q3: 0.61–0.97, Q4: >0.97) at admission, participants were stratified into four groups (Table 1). Compared with the low-LAR group (Q1), the high-LAR group (Q4) exhibited a significantly faster HR, lower SBP and MAP. Laboratory test results indicated that the high-LAR group had higher levels of WBC, RDW, anion gap, glucose, phosphate, potassium, ALT, AST, Cr, lactate, and longer PT and PTT. The high-LAR group was more likely to have complications such as AF, LD, and VA. Regarding disease severity scores, the high-LAR group had significantly higher SOFA, APSIII, SAPSII, and OASIS scores. Additionally, the high-LAR group showed lower usage rates of anti-heart failure medications and higher utilization rates of CRRT. Notably, both 28-day mortality (15.66% vs. 19.91% vs. 23.77% vs. 32.30%; *P* < 0.01) and 365-day mortality (22.26% vs. 27.45% vs. 30.57% vs. 40.21%; *P* < 0.01) were higher in the Q4 group compared with the other three groups, while no significant differences were observed among the Q1, Q2, and Q3 groups.

**Table 1:** Baseline characteristics of patients grouped according to LAR quartiles.

Variable	Overall (*n* = 4,242)	Q1(<0.41) (*n* = 1,060)	Q2 (0.41–0.61) (*n* = 1,060)	Q3 (0.61–0.97) (*n* = 1,060)	Q4(>0.97) (*n* = 1,062)	*P*-value
Demographics						
Age, years	72.04 ± 13.50	71.26 ± 13.36	72.06 ± 13.35	72.58 ± 13.20	72.27 ± 14.05	0.063
Male, *n* (*p* %)	2,432.00 (57.33%)	585.00 (55.19%)	620.00 (58.49%)	620.00 (58.49%)	607.00 (57.16%)	0.367
Vital signs						
HR, beats/min	91.48 ± 21.75	87.24 ± 19.32	89.88 ± 21.15	92.31 ± 21.07	96.50 ± 24.13	<0.001
RR, rate times/min	20.47 ± 6.41	20.20 ± 6.10	20.35 ± 6.27	20.45 ± 6.52	20.87 ± 6.73	0.096
SBP, mmHg	117.46 ± 25.03	121.43 ± 25.09	118.50 ± 24.18	116.44 ± 24.35	113.49 ± 25.82	<0.001
DBP, mmHg	66.96 ± 19.51	67.51 ± 18.81	67.46 ± 19.06	66.81 ± 19.66	66.07 ± 20.48	0.109
MAP, mmHg	79.73 ± 19.33	80.96 ± 18.76	80.34 ± 18.81	79.48 ± 19.45	78.16 ± 20.16	0.001
SpO_2_, %	97.00 (94.00, 100.00)	97.00 (94.00, 100.00)	97.00 (94.00, 100.00)	97.00 (94.00, 100.00)	98.00 (94.00, 100.00)	0.072
Comorbidities, *n* (%)						
AF	1,268.00 (29.89%)	254.00 (23.96%)	325.00 (30.66%)	346.00 (32.64%)	343.00 (32.30%)	<0.001
Hypertension	991.00 (23.36%)	267.00 (25.19%)	233.00 (21.98%)	251.00 (23.68%)	240.00 (22.60%)	0.32
LD	678.00 (15.98%)	89.00 (8.40%)	131.00 (12.36%)	182.00 (17.17%)	276.00 (25.99%)	<0.001
CKD	1,505.00 (35.48%)	402.00 (37.92%)	382.00 (36.04%)	354.00 (33.40%)	367.00 (34.56%)	0.15
MT	656.00 (15.46%)	168.00 (15.85%)	167.00 (15.75%)	148.00 (13.96%)	173.00 (16.29%)	0.462
Diabetes	1,774.00 (41.82%)	444.00 (41.89%)	439.00 (41.42%)	429.00 (40.47%)	462.00 (43.50%)	0.552
AMI	837.00 (19.73%)	190.00 (17.92%)	213.00 (20.09%)	201.00 (18.96%)	233.00 (21.94%)	0.115
COPD	1,036.00 (24.42%)	311.00 (29.34%)	263.00 (24.81%)	256.00 (24.15%)	206.00 (19.40%)	<0.001
VA	515.00 (12.14%)	92.00 (8.68%)	138.00 (13.02%)	131.00 (12.36%)	154.00 (14.50%)	<0.001
Medication, *n* (%)						
CCB	723.00 (17.04%)	212.00 (20.00%)	189.00 (17.83%)	173.00 (16.32%)	149.00 (14.03%)	0.003
Diuretics	1,324.00 (31.21%)	370.00 (34.91%)	319.00 (30.09%)	362.00 (34.15%)	273.00 (25.71%)	<0.001
Beta-blockers	390.00 (9.19%)	91.00 (8.58%)	102.00 (9.62%)	100.00 (9.43%)	97.00 (9.13%)	0.854
ACEIs/ARBs	1,390.00 (32.77%)	375.00 (35.38%)	363.00 (34.25%)	361.00 (34.06%)	291.00 (27.40%)	<0.001
GC	1,419.00 (33.45%)	340.00 (32.08%)	350.00 (33.02%)	370.00 (34.91%)	359.00 (33.80%)	0.561
CRRT	578.00 (13.63%)	112.00 (10.57%)	122.00 (11.51%)	126.00 (11.89%)	218.00 (20.53%)	<0.001
Vasopressor	3,189.00 (75.18%)	709.00 (66.89%)	743.00 (70.09%)	817.00 (77.08%)	920.00 (86.63%)	<0.001
Score system, points						
SOFA	6.87 ± 3.51	5.65 ± 2.94	6.24 ± 3.15	6.95 ± 3.35	8.65 ± 3.82	<0.001
APSIII	56.61 ± 21.01	49.74 ± 17.21	52.45 ± 18.05	56.87 ± 19.79	67.35 ± 23.94	<0.001
SAPSII	44.75 ± 13.93	40.13 ± 12.05	42.33 ± 12.53	45.17 ± 13.16	51.35 ± 15.17	<0.001
OASIS	35.59 ± 8.77	33.46 ± 8.39	34.33 ± 8.13	35.95 ± 8.55	38.63 ± 9.08	<0.001
CCI	7.05 ± 2.65	6.86 ± 2.50	7.08 ± 2.72	7.08 ± 2.68	7.16 ± 2.69	0.093
Laboratory data						
Albumin, mg/dl	2.99 ± 0.59	3.26 ± 0.50	3.07 ± 0.54	2.92 ± 0.57	2.72 ± 0.60	<0.001
Calcium, mg/dl	8.28 ± 0.88	8.42 ± 0.77	8.32 ± 0.79	8.25 ± 0.90	8.14 ± 1.01	<0.001
Chloride, mEq/L	102.46 ± 7.21	101.87 ± 6.81	102.21 ± 7.15	102.88 ± 7.23	102.88 ± 7.60	<0.001
Potassium, mEq/L	4.36 ± 0.83	4.30 ± 0.76	4.31 ± 0.79	4.37 ± 0.82	4.45 ± 0.94	0.002
Sodium, mEq/L	137.91 ± 5.90	137.91 ± 5.56	137.85 ± 6.00	137.93 ± 5.63	137.95 ± 6.38	0.858
Bicarbonate, mEq/L	22.35 ± 5.44	24.20 ± 5.49	23.20 ± 5.21	22.42 ± 4.62	19.60 ± 5.31	<0.001
Hemoglobin, g/dl	10.41 ± 2.31	10.30 ± 2.20	10.45 ± 2.27	10.56 ± 2.23	10.34 ± 2.51	0.04
Platelet, K/µl	188.00 (132.00, 258.00)	196.50 (146.00, 263.00)	191.00 (134.50, 265.00)	190.50 (133.00, 253.00)	171.00 (116.00, 246.00)	<0.001
RDW, %	15.30 (14.00, 17.00)	15.10 (14.00, 16.70)	15.20 (14.00, 17.00)	15.20 (14.00, 16.85)	15.50 (14.10, 17.40)	0.002
RBC, K/µl	3.52 ± 0.81	3.49 ± 0.75	3.55 ± 0.79	3.57 ± 0.79	3.48 ± 0.88	0.047
WBC, K/µl	12.30 (8.60, 17.30)	10.60 (7.70, 14.10)	11.75 (8.50, 16.45)	13.40 (9.20, 18.60)	14.35 (9.70, 20.50)	<0.001
Anion gap, mmol/L	15.00 (13.00, 18.00)	14.00 (12.00, 17.00)	15.00 (12.00, 17.00)	15.00 (12.00, 18.00)	17.00 (14.00, 21.00)	<0.001
Glucose, g/dl	138.00 (110.00, 183.00)	130.00 (105.50, 165.00)	134.00 (108.00, 169.00)	139.00 (112.00, 187.00)	155.00 (114.00, 221.00)	<0.001
Phosphate, mg/dl	3.90 (3.10, 4.90)	3.80 (3.20, 4.80)	3.90 (3.10, 4.80)	3.80 (3.00, 4.70)	4.10 (3.20, 5.50)	<0.001
ALT, IU/L	28.00 (16.00, 69.00)	22.00 (14.00, 39.00)	27.00 (16.00, 62.50)	28.00 (16.00, 65.00)	41.50 (19.00, 164.00)	<0.001
AST, IU/L	43.00 (25.00, 109.00)	33.00 (22.00, 59.00)	40.00 (24.00, 84.50)	44.00 (26.00, 107.00)	75.50 (34.00, 291.00)	<0.001
TBIL, mg/dl	0.70 (0.40, 1.30)	0.60 (0.40, 0.90)	0.70 (0.40, 1.10)	0.70 (0.40, 1.40)	0.90 (0.50, 1.90)	<0.001
Cr, mg/dl	1.40 (1.00, 2.30)	1.30 (0.90, 2.30)	1.40 (1.00, 2.25)	1.30 (0.90, 2.10)	1.60 (1.00, 2.40)	<0.001
BUN, mg/dl	31.00 (19.00, 50.00)	30.00 (19.00, 50.00)	30.00 (20.00, 50.50)	29.50 (19.00, 49.00)	32.00 (20.00, 49.00)	0.665
Lactate, mmol/L	1.80 (1.20, 2.80)	1.00 (0.80, 1.20)	1.50 (1.30, 1.70)	2.20 (1.90, 2.50)	3.90 (3.20, 5.80)	<0.001
PCO_2_, mmHg	41.00 (35.00, 49.00)	43.00 (37.00, 52.00)	42.00 (36.00, 49.00)	41.00 (35.00, 48.00)	39.00 (33.00, 47.00)	<0.001
PO_2_, mmHg	83.00 (48.00, 161.00)	90.00 (56.00, 161.00)	80.00 (48.00, 141.00)	82.00 (46.00, 161.00)	80.00 (44.00, 178.00)	<0.001
pH	7.35 ± 0.10	7.36 ± 0.09	7.37 ± 0.09	7.37 ± 0.09	7.32 ± 0.12	<0.001
INR	1.40 (1.20, 1.80)	1.30 (1.10, 1.50)	1.40 (1.20, 1.70)	1.40 (1.20, 1.70)	1.60 (1.30, 2.00)	<0.001
PT, s	15.20 (13.20, 19.00)	14.00 (12.60, 16.80)	15.00 (13.10–18.80)	15.30 (13.30, 18.70)	17.00 (14.30, 21.80)	<0.001
PTT, s	33.10 (28.40, 42.90)	32.20 (28.00–40.60)	32.60 (28.20–42.20)	32.60 (28.20, 42.30)	35.25 (29.40, 45.20)	<0.001
Outcome						
28-day mortality, *n* (%)	972.00 (22.91%)	166.00 (15.66%)	211.00 (19.91%)	252.00 (23.77%)	343.00 (32.30%)	<0.001
365-day mortality, *n* (%)	1,278.00 (30.13%)	236.00 (22.26%)	291.00 (27.45%)	325.00 (30.66%)	426.00 (40.11%)	<0.001

ACEIs/ARBs, angiotensin-converting enzyme inhibitors/angiotensin receptor blockers; AF, atrial fibrillation; AMI, acute myocardial infarct; ALT, alanine aminotransferase; APSIII, Acute Physiology Score III; AST, asparate aminotransferase; BUN, blood urea nitrogen; CCB, calcium channel blocker; CCI, Charlson Comorbidity Index; CKD, chronic kidney disease; COPD, chronic obstructive pulmonary disease; Cr:creatinine; CRRT, continuous renal replacement therapy; DBP, diastolic blood pressure; GC, glucocorticoid; HR, heart rate; INR: international normalized ratio; LD, liver disease; MAP, mean arterial pressure; MT, malignant tumor; OASIS, Oxford Acute Severity of Illness Score; PT, prothrombin time; PTT, partial prothrombin time; RBC, red blood cell; RDW: red cell distribution width; RR, respiratory rate; SAPSII, Simplified Acute Physiology Score II; SBP, systolic blood pressure; TBIL, total bilirubin; SOFA, Sequential Organ Failure Assessment; SpO_2_, saturation of peripheral oxygen; VA, ventricular arrhythmia; WBC, white blood cell.

### 3.2 Associations of LAR and all-cause mortality

**Figure 2:**
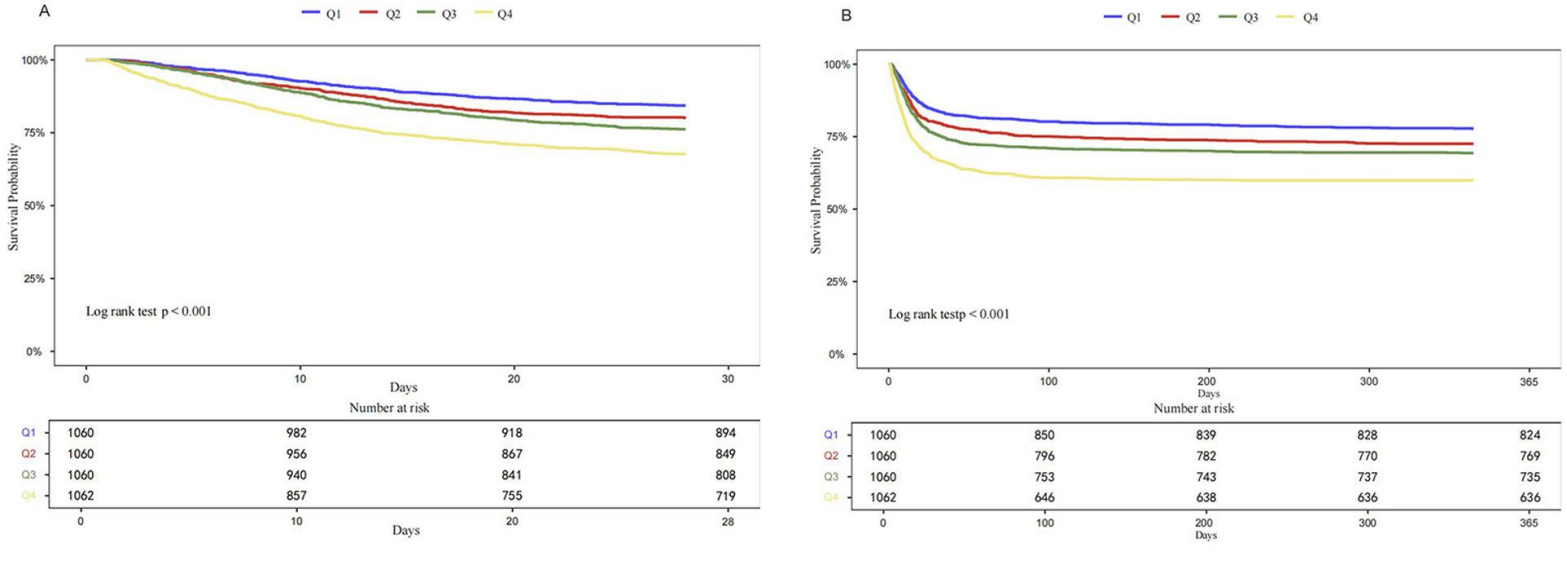
Kaplan–Meier curves for 28-days all-cause mortality **(A)** and 365-day all-cause mortality **(B)** according to quartile of LAR.

**Figure 3:**
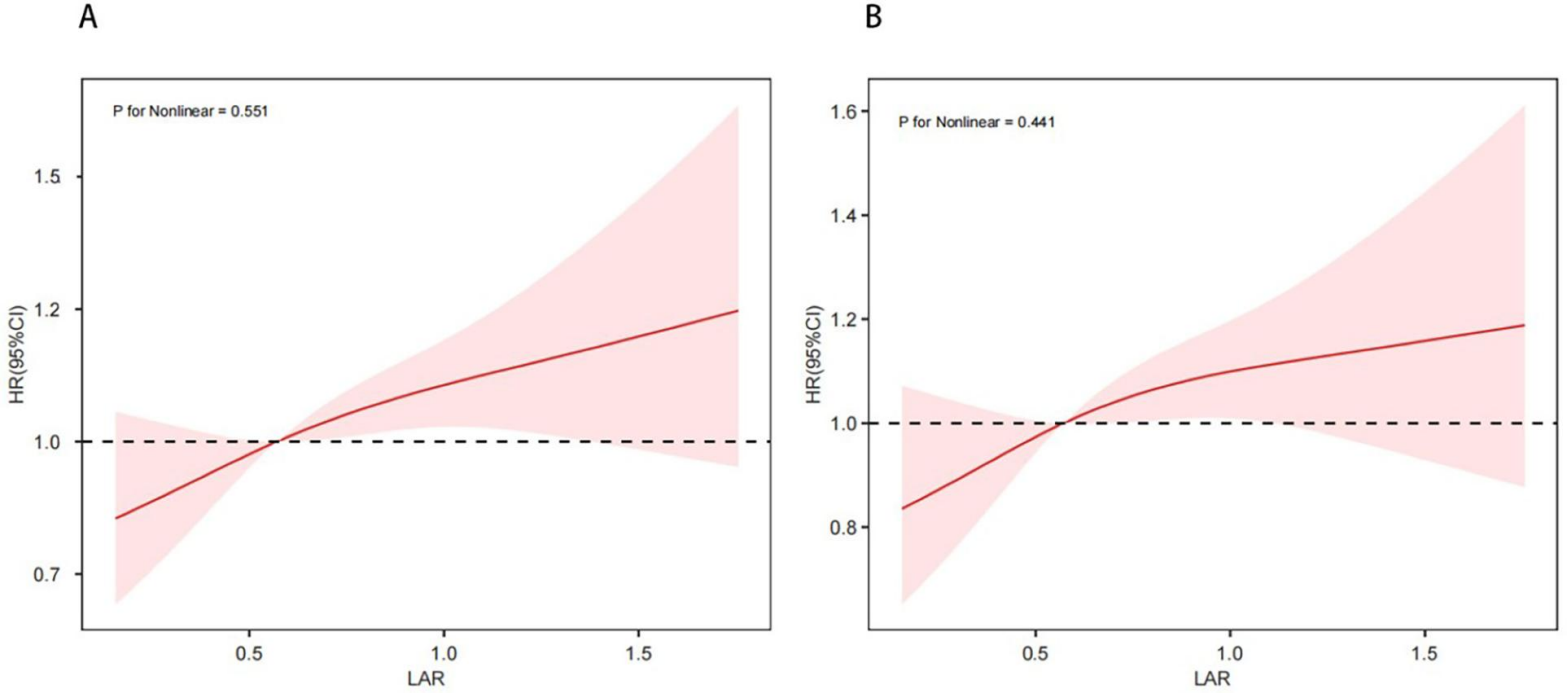
Restricted cubic spline curve for 28-day mortality **(A)** and 365-day mortality **(B)**.

Kaplan–Meier survival analysis was used to evaluate the cumulative incidence of all-cause mortality across the four groups stratified by LAR quartiles. The Kaplan–Meier curves shows that when the observation outcome was 28 days, the survival probability of Q4 group was lower compared with groups Q1, Q2 and Q3 groups (log-rank *P* < 0.001) (Figure 2A). A similar trend was observed when the observation period was extended to 365 days (log-rank *P* < 0.001) (Figure 2B). Further analysis using RCS showed a significant linear and positive association between LAR and both 28-day mortality (Figure 3A) and 365-day mortality (Figure 3B) in patients with HF complicated by sepsis (all *P* for nonlinear >0.05).

Additionally, Cox regression analyses were performed to assess the associations between LAR and short-term and long-term mortality. After full adjustment, LAR remained independently associated with an increased risk of both 28-day mortality (HR: 1.101, 95% CI: 1.005–1.205) and 365-day mortality (HR: 1.125, 95% CI: 1.039–1.218). When LAR was analyzed as a categorical variable, the highest quartile (Q4) remained significantly associated with both 28-day mortality (Q4 vs. Q1: HR: 1.313, 95% CI: 1.063–1.622) and 365-day mortality (Q4 vs. Q1: HR: 1.310, 95% CI: 1.092–1.571) (Table 2).

**Table 2:** The associations of LAR with all-cause mortality in HF patients with sepsis.

28-day mortality	Model 1		Model 2		Model 3		Model 4	
	HR (95%CI)	*P* value	HR (95%CI)	*P* value	HR (95%CI)	*P* value	HR (95%CI)	*P* value
Per unit of LAR	1.352 (1.282, 1.425)	<0.001	1.330 (1.261, 1.404)	<0.001	1.273 (1.176, 1.377)	<0.001	1.101 (1.005, 1.205)	0.038
Quartile								
Q1	1.0		1.0		1.0		1.0	
Q2	1.308 (1.068, 1.603)	0.01	1.267 (1.033, 1.553)	0.023	1.256 (1.023, 1.542)	0.029	1.144 (0.931, 1.406)	0.201
Q3	1.589 (1.306, 1.932)	<0.001	1.513 (1.243, 1.841)	<0.001	1.526 (1.248, 1.865)	<0.001	1.211 (0.987, 1.487)	0.067
Q4	2.351 (1.953, 2.830)	<0.001	2.207 (1.830, 2.660)	<0.001	1.971 (1.609, 2.415)	<0.001	1.313 (1.063, 1.622)	0.012
*P* for trend	<0.001		<0.001		<0.001		0.012	
365-day mortality	HR (95%CI)	*P* value	HR (95%CI)	*P* value	HR (95%CI)	*P* value	HR (95%CI)	*P* value
Per unit of LAR	1.328 (1.266, 1.394)	<0.001	1.306 (1.244, 1.372)	<0.001	1.288 (1.202, 1.381)	<0.001	1.125 (1.039, 1.218)	0.004
Quartile								
Q1	1.0		1.0		1.0		1.0	
Q2	1.279 (1.077, 1.518)	0.005	1.241 (1.045, 1.474)	0.014	1.237 (1.040, 1.471)	0.016	1.132 (0.951, 1.347)	0.164
Q3	1.466 (1.240, 1.734)	<0.001	1.404 (1.186, 1.660)	<0.001	1.441 (1.213, 1.711)	<0.001	1.172 (0.984, 1.396)	0.075
Q4	2.115 (1.804, 2.479)	<0.001	1.994 (1.698, 2.341)	<0.001	1.860 (1.562, 2.216)	<0.001	1.310 (1.092, 1.571)	0.004
*P* for trend	<0.001		<0.001		<0.001		0.004	

Model 1: unadjusted. Model 2: adjusted for Age, RR, SBP, SpO_2_. Model 3: adjusted for Age, RR, SBP, SpO_2_, WBC, RDW, anion gap, chloride, potassium, phosphate, bicarbonate, PTT, AST, TBIL, Cr, BUN, PCO_2_, PO_2_, pH. Model 4: adjusted for Age, RR, SBP, SpO_2_, WBC, RDW, anion gap, chloride, potassium, phosphate, bicarbonate, PTT, AST, TBIL, Cr, BUN, PCO_2_, PO_2_, pH, ACEIs/ARBs, Beta-blockers, CCB, CRRT, Vasopressor, Diuretics, GC, MT, CKD, AF, COPD, VA, Hypertension, LD, AMI, APSIII, OASIS, SAPSII, SOFA, CCI. Abbreviations as in Table 1.

### 3.3 Subgroup analysis

**Figure 4:**
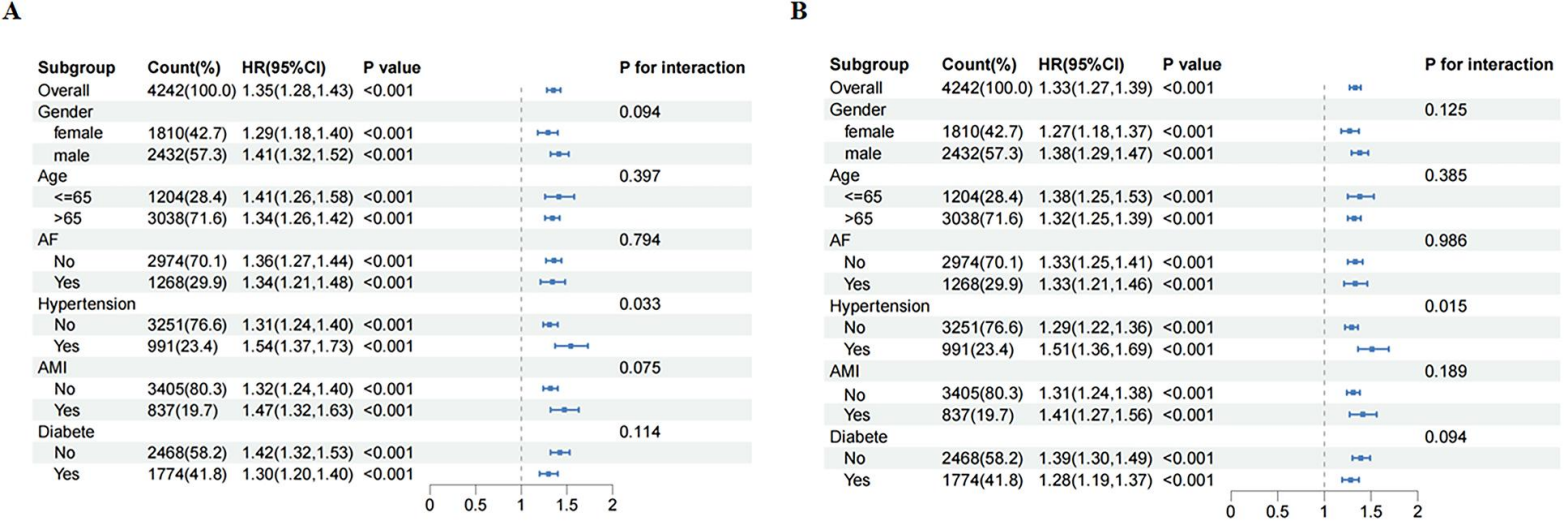
Subgroup analysis for risk of the 28-day **(A)** and 365-day **(B)** all cause mortality according to LAR.

When 28-day mortality was taken as the observation outcome, no significant interaction effects were observed across subgroups stratified by age, sex, AF, diabetes, and AMI. However, a significant interaction was identified in the hypertension subgroup (*P* for interaction = 0.033) (Figure 4A). A similar interaction was observed for 365-day mortality among hypertensive patients (*P* for interaction = 0.015; Figure 4B).

### 3.4 Sensitivity analysis

To evaluate the robustness of the results of this study, we excluded observations with missing rates below 20% and conducted a sensitivity analysis (Supplementary Table S4). Meanwhile, to minimize bias, our study excluded cases of LVEF deletion and conducted a sensitivity analysis to confirm the robustness of the research results (Supplementary Table S5). The findings from these sensitivity analyses demonstrated consistent results with the primary multivariate Cox regression model, indicating strong robustness of the main study outcomes.

## 4 Discussion

This study demonstrates that LAR is a valuable prognostic indicator for adverse outcomes in patients with HF complicated by sepsis. It enables rapid identification of high-risk ICU patients and is easily calculated from routine admission laboratory data, making it more practical than many existing tools. As a continuous predictor, LAR shows a significant linear association with both short-term and long-term mortality, with the highest risk observed in the top quartile. Subgroup analysis indicated that the association between LAR and mortality was modified by hypertension, with a pronounced difference in risk between hypertensive and non-hypertensive patients. Taken together, these findings highlight LAR as more than a general biomarker, positioning it as a useful tool for assessing patients with severe HF.

This work is particularly timely given the clinical challenges of managing HF in the setting of severe infections. Sepsis is a well-recognized trigger of disease progression in HF, and affected patients experience markedly higher ICU admission and mortality rates compared with those without sepsis. While several comprehensive risk scores exist for critically ill patients, ICU clinicians need simpler and more reliable stratification methods tailored to this high-risk group. Our findings suggest that LAR can provide meaningful risk differentiation in this population. Incorporating LAR into clinical practice may enhance risk prediction, guide treatment strategies, and ultimately improve outcomes while reducing healthcare costs.

Previous studies have consistently shown the prognostic value of LAR in sepsis and other cardiovascular diseases. LAR can also predict mortality in patients with sepsis. For example, Shin et al. proposed that LAR offers a threefold advantage in assessing the prognosis of patients with severe sepsis (22). First, LAR can predict mortality more accurately than lactic acid levels alone. Second, for patients with normal or moderate lactate levels, LAR can be used as an auxiliary indicator to effectively identify potentially high-risk individuals. Finally, LAR can significantly improve the predictive efficacy of hyperlactatemia in patients with abnormal lactic acid metabolism due to liver and kidney dysfunction. Similarly, Huang et al. demonstrated its predictive utility in AF patients admitted to the ICU (13), and Wang et al. confirmed its value in AMI (15).

Our research extended the above findings to the population with HF complicated by sepsis. We found that the association between LAR and mortality shows a continuous linear positive correlation, and it is statistically significant as a continuous predictor. The high risk in the Q4 group is a natural extension of this linear relationship, reflecting the high-risk characteristics of extreme LAR values rather than a threshold effect. Sensitivity analysis supported the robustness of these findings.

The pathophysiological basis for these associations can be explained as follows. Sepsis involves tissue hypoxia and increased aerobic glycolysis secondary to stress response activation (adrenergic stimulation) (23). In critically ill HF patients, impaired cardiac function or extensive diuretic use can exacerbate tissue hypoperfusion and anaerobic metabolism, elevating lactate levels (12). Consequently, lactate levels may be elevated to a greater degree in patients with both HF and sepsis than in those with either condition alone. As the most abundant protein in plasma, serum albumin is widely used in clinical disease monitoring (24). Previous studies report hypoalbuminemia in approximately 14% of acute HF patients, correlating with poorer prognosis (25). In our cohort, high LAR reflected both elevated lactate and hypoalbuminemia, which collectively heighten the risk of all-cause death in severe HF patients complicated by sepsis.

The interaction observed in hypertensive subgroups may involve several mechanisms: endothelial dysfunction exacerbating oxidative stress in HF complicated by sepsis, microcirculatory impairment, activation of the renin-angiotensin system (RAS), and patterns of RAS inhibitor use. Chronic hypertension impairs vascular endothelial function, promoting elevated inflammatory mediators that may exacerbate systemic inflammation during sepsis (26, 27). Sun et al. reported that hypertension increases sepsis-related 28-day mortality risk, because hypertension-associated endothelial dysfunction and inflammatory cascade may contribute to this elevated mortality risk in septic patients (28). Additionally, sepsis-induced microcirculatory alterations are complex and heterogeneous, in which hypoperfused capillaries can compromise tissue oxygenation and contribute to multi-organ dysfunction (26). Maintaining higher MAP levels could potentially improve capillary perfusion pressure in such patients, partially compensating for impaired microcirculatory regulation. Lee et al. observed improved prognosis when MAP was maintained between 75 and 85 mmHg in chronically hypertensive sepsis patients (29). Furthermore, the Renin- Angiotensin-Aldosterone System (RAAS) is activated in sepsis. Angiotensin II (Ang II), the primary effector of RAAS, functions as a key inflammatory mediator associated with organ failure and mortality (30). Recent observational cohorts suggest that RAS inhibitors may reduce all-cause mortality in sepsis patients with concurrent hypertension or HF (31, 32).

However, we observed an interesting phenomenon: the overall utilization rate of RAS inhibitors in the high LAR group in Table 1 was relatively low (Q4: 27.40% vs. Q1: 35.38%, *P* < 0.001). This apparent contradiction prompted further investigation into the hypertension subgroup interaction. To determine whether similar patterns existed specifically among hypertensive patients, we conducted baseline comparisons (Supplementary Table S6). These revealed significantly higher RAS inhibitor usage in hypertensive vs. non-hypertensive patients (44.70% vs. 29.13%, *P* < 0.001), while beta-blockers usage showed no difference (9.18% vs. 9.20%, *P* = 0.989). As previously mentioned, observational studies suggest RAS inhibitors may reduce mortality risk. We hypothesize that the lower usage in high LAR patients likely reflects clinicians’ concerns about prescribing these medications to patients with more severe tissue hypoperfusion and hepatic/renal dysfunction (indicated by high LAR), particularly given risks of hypotension or renal function deterioration. Consequently, restricted RAS inhibitor use in high LAR patients could attenuate their potential protective effect in hypertensive individuals, thereby amplifying mortality risk and potentially explaining the stronger LAR-mortality association observed in this subgroup. Importantly, beta-blockers usage patterns showed no significant association with this interaction.

It is undeniable that heterogeneity exists among study populations, warranting further investigation in future studies. Current research on clinical subphenotypes of sepsis is central to patient heterogeneity studies, as identifying these subphenotypes is critical for developing tailored treatment strategies in this population. The research by Yang et al. establishes a methodological paradigm for sepsis heterogeneity research (33). Adapting their framework to LAR investigations will advance our understanding of this biomarker's prognostic value across diverse patient subpopulations, ultimately enabling more precise risk stratification and therapeutic decision-making. Applying this approach to the high-risk population with HF complicated by sepsis is expected to improve clinical decision-making and prognosis.

Furthermore, fundamental differences exist between Gram-negative and Gram-positive bacteria in their structural composition and corresponding host responses. These pathogen-specific variations may influence prognosis in sepsis. Notably, Gram-negative infections may provoke more severe inflammatory responses than Gram-positive infections, though survival outcomes show no significant difference (34). Additionally, endotoxins from Gram-negative bacteria can elevate lactatemia through increased lactate production (35). Due to the limitations of the database and the existence of culture-negative sepsis, our study couldn't conduct subgroup analyses of sepsis patients infected with different types of bacteria.

Although our research results strongly demonstrated the prognostic value of LAR in HF complicated by sepsis using a single-center database, larger-scale studies are warranted to validate the universal applicability of our conclusions. We also acknowledge that, given the observational and retrospective design, the relationship between LAR and outcomes should be interpreted as associative rather than causal. Therefore, in the future, our research needs to further explore the causal relationship between LAR and outcomes. At present, in addition to traditional randomized controlled trials (RCTs), emerging methods like Mendelian randomization (MR), machine learning (ML), propensity score matching (PSM), target trial emulation (TTE) offer new causal inference frameworks using observational data. Recently, the TTE framework developed by Jie Yang et al. provides a methodological foundation for investigating causal relationships between exposures and outcomes (36). By integrating TTE framework with other statistical methods, researchers can move beyond the inherent limitations of conventional observational analyses and generate more robust evidence regarding potential causal links. Future work should apply these methods to test whether LAR is causally related to all-cause mortality in this population.

Moreover, LVEF, BNP and NT-proBNP serve as key biological indicators for HF classification, severity assessment, and prognosis prediction. However, due to high rates of missing data (LVEF for 38.5% and NT-proBNP for 76.9%) exceeding our predefined threshold (>20%), these variables were excluded from the analysis. As a result, we were unable to classify HF phenotypes, which may follow different prognostic trajectories in sepsis. Although our findings were consistent with those from the sensitivity analysis, the absence of reliable natriuretic peptide and echocardiographic data limits the generalizability of results. Future studies that combine complete biomarkers and imaging information are needed to clarify whether the prognostic effect of LAR varies with HF phenotypes. Notably, the substantial of missingness of NT-proBNP in the MIMIC-IV database precluded meaningful analysis of this biomarker, as complete case exclusion would have caused significant sample size reduction, selection bias, and diminished statistical power.

Several limitations should be addressed in this study. First, the single-center retrospective design may introduce selection bias and limit the generalizability of our findings to other populations with differing demographics or healthcare systems. Second, due to the limitations of the MIMIC-IV database, key cardiac function indicators were not collected for all patients, which could not conduct further analysis on the prognosis of patients with different types of HF. Third, although we adjusted for multiple confounders, residual confounding such as medication adherence, lifestyle behaviors and physician decisions were not available in the database, which may have influenced the predictive performance of LAR. Fourth, all hospitalized patients were from a U.S. population, thus the generalizability of the results to other populations requires further validation. Finally, our analysis cannot infer causality between LAR and mortality and lacked dynamic assessment of how changes in LAR affect prognosis.

Future studies should validate these findings across diverse populations and further explore the underlying pathophysiological mechanisms. Additionally, dynamic monitoring of LAR in critically ill patients may offer additional prognostic value. Further research should aim to clarify the role of LAR in predicting mortality among different types of HF patients complicated by sepsis. Advancing these research directions may contribute to more personalized treatment strategies for this patient population.

## 5 Conclusion

This study demonstrates that the LAR serves as a reliable and independent prognostic indicator for critically ill patients with HF complicated by sepsis. The highest LAR quartile (>0.97) is associated with a significantly elevated mortality risk in this population, enabling rapid identification of high-risk individuals and timely implementation of relevant treatments. Furthermore, within the hypertensive subgroup, the analysis revealed a higher mortality risk among hypertensive patients compared to non-hypertensive individuals. Clinicians should maintain heightened vigilance in managing these patients. In summary, LAR integrates information on metabolic dysfunction, inflammation and nutritional status, making it a valuable tool for early risk stratification and individualized treatment planning in high-risk groups. Further research should prospectively validate these findings in multicenter studies and explore the utility of monitoring LAR dynamics during hospitalization. Such efforts would enhance its clinical applicability, leading to improved prognostic precision and more tailored management strategies for patients with HF complicated by sepsis.

## Data Availability

The original contributions presented in the study are included in the article/Supplementary Material, further inquiries can be directed to the corresponding author.
