# Supplementary material for: Association of lactate to albumin ratio with short-term and long-term mortality in critically ill patients with heart failure complicated by sepsis: a retrospective study using the MIMIC-IV database
Source: Front Cardiovasc Med. 2025 Sep 23;12:1636375. doi: 10.3389/fcvm.2025.1636375 (PMC12500606; doi:10.3389/fcvm.2025.1636375)
Supplement: Supplementary file 1 [file Datasheet1.pdf]

Table S1. Missing values.

Variable Names	Missing values (%)	Variable Names	Missing values (%)
Age	0.0	Chloride	0.0
HR	0.0	Glucose	0.0
SBP	1.1	Potassium	0.1
DBP	1.2	Sodium	0.0
MAP	1.1	Phosphate	0.2
RR	0.0	ALT	5.4
SpO_2	0.0	AST	4.1
SOFA	0.0	TBIL	4.6
APSIII	0.0	Cr	0.0
SAPSII	0.0	BUN	0.0
OASIS	0.0	Lactate	0.0
CCI	0.0	PCO_2	4.2
Haemoglobin	0.5	pH	3.2
Platelets	0.5	PO_2	4.1
RDW	0.6	INR	2.2
RBC	0.5	PT	2.2
WBC	0.5	PTT	2.7
Albumin	0.0	Bicarbonate	0.0
Anion gap	0.0	LVEF	38.5
Calcium	0.2	NT-proBNP	76.9

Abbreviations as in Table 1.

Table S2. Univariate COX regression analysis of 28-day mortality and 365-day mortality of the patients.

Variables	28-day mortality		365-day mortality	
	HR (95%CI)	p-value	HR (95%CI)	p-value
ACEIs/ARBs	0.252 (0.208,0.304)	0.001	0.376 (0.326,0.434)	0.001
AF	1.231 (1.078,1.406)	0.002	1.275 (1.136,1.431)	0.001
Anion gap	1.041 (1.029,1.054)	0.001	1.035 (1.024,1.046)	0.001
Age	1.023 (1.018,1.029)	0.001	1.018 (1.014,1.023)	0.001
Albumin	0.634 (0.570,0.706)	0.001	0.651 (0.593,0.715)	0.001
ALT	1.008 (1.002,1.012)	0.028	1.005 (0.982,1.020)	0.055
AMI	1.266 (1.090,1.470)	0.002	1.239 (1.087,1.413)	0.001
APSIII	1.021 (1.0191.024)	0.001	1.020 (1.018,1.022)	0.001
AST	1.001 (0.967,1.015)	0.001	1.032 (1.001,1.070)	0.004
Beta blockers	0.293 (0.205,0.419)	0.001	0.397 (0.303,0.518)	0.001
Bicarbonate	0.982 (0.970,0.994)	0.002	0.987 (0.977,0.998)	0.016
BUN	1.009 (1.007,1.011)	0.001	1.008 (1.007,1.010)	0.001
Calcium	0.981 (0.912,1.055)	0.602	0.997 (0.936,1.062)	0.922
CCB	0.604 (0.497,0.734)	0.001	0.776 (0.665,0.907)	0.001
CCI	1.133 (1.107,1.158)	0.001	1.131 (1.109,1.154)	0.001
CKD	1.249 (1.099,1.420)	0.001	1.273 (1.139,1.424)	0.001
Chloride	0.982 (0.973,0.990)	0.001	0.978 (0.971,0.985)	0.001
COPD	1.185 (1.029,1.365)	0.019	1.162 (1.026,1.315)	0.018
Cr	1.061 (1.031,1.092)	0.001	1.066 (1.040,1.093)	0.001
CRRT	2.515 (2.177,2.905)	0.001	2.505 (2.204,2.847)	0.001
DBP	0.998 (0.995,1.001)	0.224	0.998 (0.995,1.002)	0.091
Diuretic	0.238 (0.195,0.290)	0.001	0.388 (0.336,0.448)	0.001
Diabetes	0.972 (0.856,1.105)	0.665	0.995 (0.891,1.112)	0.936

GC	1.308 (1.150,1.488)	0.001	1.422 (1.272,1.590)	0.001
Gender	1.010 (0.889,1.147)	0.878	0.992 (0.888,1.109)	0.89
Glucose	1.020 (1.002,1.035)	0.06	1.005 (0.982,1.015)	0.298
Haemoglobin	0.963 (0.937,0.99)	0.008	0.947 (0.924,0.970)	0.001
HR	1.002 (0.999,1.005)	0.137	1.002 (1.000,1.005)	0.108
Hypertension	0.755 (0.644,0.886)	0.001	0.728 (0.633,0.837)	0.001
Potassium	1.210 (1.128,1.298)	0.001	1.187 (1.116,1.263)	0.001
Lactate	1.115 (1.090,1.139)	0.001	1.108 (1.086,1.131)	0.001
LAR	1.352 (1.282,1.425)	0.001	1.328 (1.266,1.394)	0.001
LD	1.751 (1.509,2.031)	0.001	1.794 (1.575,2.042)	0.001
MAP	0.998 (0.994,1.001)	0.158	0.997 (0.994,1.000)	0.067
MT	1.255 (1.066,1.477)	0.006	1.207 (1.045,1.394)	0.011
Sodium	0.997 (0.986,1.008)	0.564	0.989 (0.980,0.998)	0.022
OASIS	1.049 (1.041,1.056)	0.001	1.040 (1.033,1.046)	0.001
Phosphate	1.183 (1.144,1.223)	0.001	1.163 (1.129,1.199)	0.001
PCO_2	1.006 (1.002,1.011)	0.007	1.005 (1.001,1.009)	0.013
pH	0.101 (0.056,0.179)	0.001	0.156 (0.093,0.262)	0.001
platelets	1.052 (0.982,1.221)	0.402	1.052 (0.999,1.125)	0.574
PO_2	0.998 (0.997,0.999)	0.001	0.999 (0.998,0.999)	0.001
INR	1.079 (1.039, 1.121)	0.001	1.079 (1.044, 1.116)	0.001
PT	1.009 (1.005,1.013)	0.001	1.009 (1.005,1.012)	0.001
PTT	1.003 (1.001,1.005)	0.006	1.003 (1.001,1.004)	0.004
RBC	0.892 (0.824,0.965)	0.005	0.854 (0.797,0.915)	0.001
RDW	1.110 (1.087,1.133)	0.001	1.119 (1.099,1.139)	0.001
RR	1.013 (1.004,1.023)	0.006	1.012 (1.004,1.021)	0.004
SAPSII	1.035 (1.031,1.040)	0.001	1.033 (1.029,1.037)	0.001

SBP	0.996 (0.993,0.999)	0.002	0.996 (0.994,0.998)	0.001
SOFA	1.108 (1.090,1.127)	0.001	1.092 (1.076,1.108)	0.001
SpO_2	0.978 (0.967,0.990)	0.001	0.979 (0.969,0.989)	0.001
TBIL	1.062 (1.044,1.079)	0.001	1.060 (1.044,1.077)	0.001
VA	1.401 (1.178,1.665)	0.001	1.452 (1.250,1.688)	0.001
Vasopressor	2.571 (2.123,3.112)	0.001	2.251 (1.924,2.633)	0.001
WBC	1.014 (1.009,1.019)	0.001	1.012 (1.007,1.017)	0.001

Abbreviations : ACEIs/ARBs: angiotensin-converting enzyme inhibitors/angiotensin receptor blockers; AF : atrial fibrillation; AMI : acute myocardial infarct; ALT : alanine aminotransferase; APSIII : Acute Physiology Score III; AST : asparate aminotransferase; BUN : blood urea nitrogen ; CCB : calcium channel blocker; CCI : Charlson Comorbidity Index; CKD : chronic kidney disease; COPD : chronic obstructive pulmonary disease; Cr :creatinine; CRRT : continuous renal replacement therapy; DBP : diastolic blood pressure; GC : glucocorticoid; HR : heart rate; LAR : lactate to albumin ratio; LD : liver disease; MAP : mean arterial pressure; MT : malignant tumor; OASIS : Oxford Acute Severity of Illness Score; PT : prothrombin time; PTT : partial prothrombin time ; RBC : red blood cell; RDW: red cell distribution width; RR : respiratory rate; SAPSII : Simplified Acute Physiology Score II; SBP : systolic blood pressure; TBIL : total bilirubin; SOFA : Sequential Organ Failure Assessment; SpO_2 : saturation of peripheral oxygen; VA : ventricular arrhythmia; WBC : white blood cell.

Table S3. The VIF value of the variable.

Variable	VIF	Variables	VIF	Variables	VIF
Age	1.66	OASIS	2.47	Potassium	1.43
SBP	1.11	CCI	2.01	Phosphate	2.40
RR	1.16	CCB	1.05	AST	1.31
SpO_2	1.11	Diuretics	1.05	TBIL	1.41
AF	1.24	Beta blockers	1.03	Cr	2.34
Hypertension	1.30	GC	1.10	BUN	2.19
LD	1.38	Hemoglobin	6.80	Lactate	3.81
CKD	1.67	RDW	1.46	PCO_2	3.93
AMI	1.20	RBC	6.30	pH	4.34
COPD	1.10	WBC	1.10	PO_2	1.27
SOFA	2.69	Albumin	1.47	INR	14.65
APSIII	3.79	Anion gap	3.74	PT	14.85
SAPSII	4.53	Chloride	1.70	PTT	1.13
CRRT	1.42	Bicarbonate	4.93	MT	1.14
VA	1.09	Vasopressor	1.17	ACEIs/ARBs	1.09

Abbreviations : ACEIs/ARBs: angiotensin-converting enzyme inhibitors/angiotensin receptor blockers; AF : atrial fibrillation; AMI : acute myocardial infarct; ALT : alanine aminotransferase; APSIII : Acute Physiology Score III; AST : asparate aminotransferase; BUN : blood urea nitrogen ; CCB : calcium channel blocker; CCI : Charlson Comorbidity Index; CKD : chronic kidney disease; COPD : chronic obstructive pulmonary disease; Cr :creatinine; CRRT : continuous renal replacement therapy; DBP : diastolic blood pressure; GC : glucocorticoid; HR : heart rate; LD : liver disease; MAP : mean arterial pressure; MT : malignant tumor; OASIS : Oxford Acute Severity of Illness Score; PT : prothrombin time; PTT : partial prothrombin time ; RBC : red blood cell; RDW: red cell distribution width; RR : respiratory rate; SAPSII : Simplified Acute Physiology Score II; SBP : systolic blood pressure; TBIL : total bilirubin; SOFA : Sequential Organ Failure Assessment; SpO_2 : saturation of peripheral oxygen; VA : ventricular arrhythmia; WBC : white blood cell.

Table S4. Sensitivity analysis for the elimination of lactate and albumin deficiency values.

	Model 1		Model 2		Model 3		Model 4	
**28-day mortality**	**HR (95 %CI)**	**P value**	**HR (95 %CI)**	**P value**	**HR (95 %CI)**	**P value**	**HR (95 %CI)**	**P value**
Per unit of LAR	1.321(1.240, 1.408)	<0.001	1.306(1.223, 1.394)	<0.001	1.323(1.210, 1.447)	<0.001	1.126(1.018, 1.245)	0.021
Quartile								
Q1	1.0		1.0		1.0		1.0	
Q2	1.266(1.017, 1.575)	0.035	1.222(0.982, 1.521)	0.072	1.204(0.966, 1.501)	0.099	1.089(0.872, 1.360)	0.451
Q3	1.639(1.331, 2.018)	<0.001	1.540(1.250, 1.897)	<0.001	1.574(1.272, 1.947)	<0.001	1.289(1.037, 1.603)	0.022
Q4	2.248(1.842, 2.744)	<0.001	2.087(1.707, 2.551)	<0.001	2.055(1.654, 2.554)	<0.001	1.420(1.132, 1.781)	0.002
P for trend	<0.001		<0.001		<0.001		0.011	
**365-day mortality**	**HR (95 %CI)**	**P value**	**HR (95 %CI)**	**P value**	**HR (95 %CI)**	**P value**	**HR (95 %CI)**	**P value**
Per unit of LAR	1.309(1.237, 1.386)	<0.001	1.291(1.218, 1.369)	<0.001	1.317(1.217, 1.425)	<0.001	1.129(1.034, 1.233)	0.007
Quartile								
Q1	1.0		1.0		1.0		1.0	
Q2	1.337(1.109, 1.612)	0.002	1.292(1.072, 1.558)	0.007	1.278(1.058, 1.543)	0.011	1.164(0.963, 1.408)	0.116
Q3	1.582(1.319, 1.898)	<0.001	1.497(1.247, 1.796)	<0.001	1.542(1.280, 1.858)	<0.001	1.283(1.061, 1.551)	0.01
Q4	2.147(1.804, 2.555)	<0.001	2.003(1.680, 2.388)	<0.001	1.981(1.638, 2.398)	<0.001	1.421(1.165, 1.732)	<0.001
P for trend	<0.001		<0.001		<0.001		0.009	

Model 1: unadjusted.

Model 2: adjusted for Age, RR, SBP, SpO_2.

Model 3: adjusted for Age, RR, SBP, SpO_2, WBC, RDW, anion gap, chloride, potassium, phosphate, bicarbonate, PTT, AST, TBIL, Cr, BUN, PCO_2, PO_2, pH.

Model 4: adjusted for Age, RR, SBP, SpO_2, WBC, RDW, anion gap, chloride, potassium, phosphate, bicarbonate, PTT, AST, TBIL, Cr, BUN, PCO_2, PO_2, pH, ACEIs/ARBs, Beta blockers, CCB, CRRT, Vasopressor, Diuretic, GC, MT, CKD, AF, COPD, VA, Hypertension, LD, AMI, APSIII, OASIS, SAPSII, SOFA, CCI.

Abbreviations as in Table 1.

Table S5. Sensitivity analysis of LVEF missing value elimination.

	**Model 1**		**Model 2**		**Model 3**		**Model 4**	
**28-day mortality**	**HR (95 %CI)**	**P value**	**HR (95 %CI)**	**P value**	**HR (95 %CI)**	**P value**	**HR (95 %CI)**	**P value**
Per unit of LAR	1.389(1.296, 1.488)	<0.001	1.358(1.265, 1.459)	<0.001	1.360(1.221, 1.515)	<0.001	1.247(1.095, 1.420)	<0.001
Quartile								
Q1	1.0		1.0		1.0		1.0	
Q2	1.366(1.000, 1.867)	0.050	1.303(0.953, 1.781)	0.097	1.262(0.922, 1.729)	0.146	1.144(0.834, 1.569)	0.404
Q3	1.895(1.413, 2.541)	<0.001	1.746(1.300, 2.345)	<0.001	1.712(1.268, 2.310)	<0.001	1.323(0.972, 1.801)	0.075
Q4	2.690(2.032, 3.560)	<0.001	2.459(1.853, 3.262)	<0.001	2.175(1.603, 2.951)	<0.001	1.470(1.070, 2.021)	0.018
P for trend			<0.001		<0.001		<0.001	
**365-day mortality**	**HR (95 %CI)**	**P value**	**HR (95 %CI)**	**P value**	**HR (95 %CI)**	**P value**	**HR (95 %CI)**	**P value**
Per unit of LAR	1.334(1.252, 1.422)	<0.001	1.303(1.220, 1.393)	<0.001	1.319(1.200, 1.449)	<0.001	1.199(1.072, 1.340)	<0.001
Quartile								
Q1	1.0		1.0		1.0		1.0	
Q2	1.341(1.047, 1.717)	0.020	1.284(1.002, 1.646)	0.048	1.270(0.990, 1.631)	0.060	1.170(0.910, 1.504)	0.219
Q3	1.680(1.324, 2.131)	<0.001	1.556(1.225, 1.977)	<0.001	1.574(1.234, 2.008)	<0.001	1.297(1.011, 1.663)	0.041
Q4	2.196(1.746, 2.762)	<0.001	2.012(1.596, 2.537)	<0.001	1.902(1.480, 2.443)	<0.001	1.398(1.077, 1.815)	0.012
P for trend	<0.001		<0.001		<0.001		<0.001	

Model 1: unadjusted.

Model 2: adjusted for Age, RR, SBP, MAP, SpO_2.

Model 3: adjusted for Age, RR, SBP, MAP, SpO_2, WBC, RBC, RDW, anion gap, chloride, potassium, phosphate, INR, PTT, TBIL, Cr, BUN, PO_2, pH, LVEF.

Model 4: adjusted for Age, RR, SBP, MAP, SpO_2, WBC, RBC, RDW, anion gap, chloride, potassium, phosphate, INR, PTT, TBIL, Cr, BUN, PO_2, pH, LVEF, ACEIs/ARBs, AF, Beta blockers, CRRT, Vasopressor, Diuretics, GC, CKD, VA, LD, AMI, APSIII, OASIS, SAPSII, SOFA, CCI.

Abbreviations as in Table 1.

Table S6. Baseline characteristics of the hypertension subgroup.

Variable	Overall (n = 4,242)	Non - hypertension (n = 3,251)	Hypertension(n = 991)	p-value
Age, years	72.04 ± 13.50	71.79 ± 13.70	72.87 ± 12.77	0.023
SBP, mmHg	117.46 ± 25.03	116.59 ± 24.99	120.32 ± 24.95	<0.001
DBP, mmHg	66.96 ± 19.51	67.00 ± 19.59	66.83 ± 19.28	0.802
MAP, mmHg	79.73 ± 19.33	79.81 ± 19.40	79.48 ± 19.11	0.640
SOFA, points	6.87 ± 3.51	7.07 ± 3.54	6.22 ± 3.35	<0.001
APSIII, points	56.61 ± 21.01	57.59 ± 21.15	53.37 ± 20.24	<0.001
SAPSII, points	44.75 ± 13.93	45.27 ± 14.05	43.05 ± 13.39	<0.001
OASIS, points	35.59 ± 8.77	35.66 ± 8.80	35.37 ± 8.64	0.347
CCI, points	7.05 ± 2.65	7.32 ± 2.71	6.16 ± 2.24	<0.001
ACEIs/ARBs (%)	1,390.00 (32.77%)	947.00 (29.13%)	443.00 (44.70%)	<0.001
Beta blockers (%)	390.00 (9.19%)	299.00 (9.20%)	91.00 (9.18%)	0.989

Abbreviations as in Table 1.
